# Pressurized Liquid Extraction for the Recovery of Bioactive Compounds from Seaweeds for Food Industry Application: A Review

**DOI:** 10.3390/antiox12030612

**Published:** 2023-03-01

**Authors:** Ana Perez-Vazquez, Maria Carpena, Paula Barciela, Lucia Cassani, Jesus Simal-Gandara, Miguel A. Prieto

**Affiliations:** 1 Nutrition and Bromatology Group, Department of Analytical Chemistry and Food Science, Faculty of Science, Universidade de Vigo, E32004 Ourense, Spain; 2 Centro de Investigação de Montanha (CIMO), Instituto Politécnico de Bragança, Campus de Santa Apolonia, 5300-253 Bragança, Portugal

**Keywords:** pressurized liquid extraction, seaweeds, green extraction technique, bioactive compounds, functional ingredients, food packaging, future trends

## Abstract

Seaweeds are an underutilized food in the Western world, but they are widely consumed in Asia, with China being the world’s larger producer. Seaweeds have gained attention in the food industry in recent years because of their composition, which includes polysaccharides, lipids, proteins, dietary fiber, and various bioactive compounds such as vitamins, essential minerals, phenolic compounds, and pigments. Extraction techniques, ranging from more traditional techniques such as maceration to novel technologies, are required to obtain these components. Pressurized liquid extraction (PLE) is a green technique that uses high temperatures and pressure applied in conjunction with a solvent to extract components from a solid matrix. To improve the efficiency of this technique, different parameters such as the solvent, temperature, pressure, extraction time and number of cycles should be carefully optimized. It is important to note that PLE conditions allow for the extraction of target analytes in a short-time period while using less solvent and maintaining a high yield. Moreover, the combination of PLE with other techniques has been already applied to extract compounds from different matrices, including seaweeds. In this way, the combination of PLE-SFE-CO_2_ seems to be the best option considering both the higher yields obtained and the economic feasibility of a scaling-up approximation. In addition, the food industry is interested in incorporating the compounds extracted from edible seaweeds into food packaging (including edible coating, bioplastics and bio-nanocomposites incorporated into bioplastics), food products and animal feed to improve their nutritional profile and technological properties. This review attempts to compile and analyze the current data available regarding the application of PLE in seaweeds to determine the use of this extraction technique as a method to obtain active compounds of interest for food industry application.

## 1. Introduction

### 1.1. Seaweeds as a Bioactive Compound Matrix

Seaweeds, also known as macroalgae, are eukaryotic, photosynthetic, pluricellular organisms found in the marine environment. They are divided into three groups: green (Chlorophyta), brown (Phaeophyta) and red algae (Rhodophyta). They are widely consumed in Asia, with China being the world’s largest producer [1]. In recent decades, Western countries have become interested in seaweeds due to their high nutritional value [2]. Seaweeds are distinguished by their high quality profile of lipids, proteins, essential minerals, phenolic compounds and pigments. Several species, for example, such as *Palmaria palmata*, *Vertebrata lanosa* and *Enteromorpha intestinalis*, have been reported to have high quality profiles of essential amino acids and lipids when compared to other food matrices such as rice, corn, or wheat. Seaweed are an interesting matrix for the industry due to the bioactive compounds and the hydrocolloids found in them [3]. It is important to note that the species, harvest season, and eco-habitat are all factors that influence the composition of seaweed [2]. Aside from their nutritional value, some compounds found in seaweeds have different technological and biochemical properties that can be used in the food industry, either to improve the food process or to increase the nutritional value of food products. The industrial functionality as well as the principal components and bioactive properties of seaweed compounds will be briefly described to demonstrate the potential benefits of their incorporation into food products.

Seaweed carbohydrates (CH) are classified in hydrocolloids (which include carrageenan, alginates, fucoidans and laminarin) and phycocolloids (agar being the most relevant example). These carbohydrates typically account for 4–76% of the dried weight (DW), with *Ulva lactuca* reporting an 65% of DW as one of the major CH contents reported in seaweeds [4]. The lipidic content of these organisms is typically less than 5% [3], but they have been reported to present a high-quality profile of fatty acids (FAs), which may vary between 1–5% [5] and 1–8% [6]. For instance, the FA content of *Laurencia filiformis*, *Cystoseira baccata* and *Fucus vesiculosus* is 6.2%, 6.7% and 6.6%, respectively, which proves that algae genera influence the FA content. Moreover, brown and red algae have higher lipid content than green algae [7]. Despite having a lower FA content than microalgae, seaweeds are interesting to the food industry for several reasons. For example, seaweed treatment is simpler than microalgae treatment, and seaweeds contain a significant amount of unsaturated fatty acids (USFA) [7]. Protein values range between 3 and 47% with brown algae having the lowest DW of this compound. The most important seaweed protein for the food industry is lectin. Because lectin is a glycoprotein with carbohydrate-binding properties, it can agglutinate yeasts, tumor cells and erythrocytes. Because lectin has antimicrobial, antitumor and antiviral activity, it could be used as a functional ingredient [4]. The main micronutrients in algae are inorganic minerals and vitamins. These organisms are high in potassium, sodium, magnesium and calcium, as well as vitamins A, B and E, and particularly vitamin B12.

Certain organisms, including algae, produce secondary metabolites as defensive and/or adaptive responses to environmental stresses. Among these compounds, pigments and phenolic compounds are the most investigated. Regarding pigments, chlorophylls, carotenoids and phycobilins are the three major classes of photosynthetic pigments in seaweeds. Carotenoids have higher industrial value due to their ability to provide color naturally as well as their bioactive function. Carotenoids (β-carotene, lutein and astaxanthin) have antioxidant activity, immune system effects and can help to prevent cardiovascular diseases and non-alcoholic fatty liver diseases [8]. Fucoxanthin, the pigment found primarily in brown algae has attracted industry attention in the recent years due to a wide range of biological properties that may be of interest to the food and nutraceutical industries [8]. Seaweed polyphenols have also been studied, with phlorotannins being the most extensively studied. Phlorotannins are polymers and oligomers composed of several phloroglucinol units linked in different ways [9,10]. These highly hydrophilic secondary metabolites are only produced by brown algae and range in molecular size from 162 Da to 650 kDa [11,12]. Phlorotannins have been linked to several biological functions including antioxidant, anticoagulant, antibacterial, anti-inflammatory and anti-diabetic activities [12]. Therefore, the incorporation of these compounds in food formulations may be noteworthy.

### 1.2. Extraction Techniques Applied in Seaweeds Matrices

The first step in extracting target compounds from any matrix is extraction. A solvent that can penetrate the solid matrix is required for this process so the target compound can be dissolved in and extracted, and the solute of interest can be separated from the raw material [13]. Different extraction techniques have been used to release the previously mentioned target compounds. The extraction methodologies can be classified in two groups: traditional extraction techniques and new extraction techniques. Traditional methods are maceration, percolation and reflux extraction. In these techniques, an organic solvent is usually used, and large volumes and a long time of extraction are needed. These drawbacks motivated the search for new alternatives to traditional extraction techniques, which is usually related to the green revolution concept. Food technologies for preservation, processing, extraction and analysis have evolved from those conventional procedures to more innovative and environmentally friendly processes by reducing fossil energy use and hazardous solvents while avoiding water loss and residues generation. Therefore, the design of green and sustainable processes, and particularly, green extraction processes, remains a hot topic in the food industry [14].

Maceration is a solid-extraction technique characterized by its low cost and simple equipment requirements. The solubility of the target compound is determined by agitation and temperature in this technique. Moreover, the protocols used in this process are easily adaptable to obtain a wide range of compounds of interest. This is possible because different solvents, temperatures and agitation conditions can be selected, increasing mass transfer selectivity and efficiency. Unfortunately, several cycles of filtration or centrifugation are required to separate the compounds from the matrix [15]. Percolation is an extraction technique that works continuously. As a result, the saturated solvent is continuously replaced by a fresh one, increasing efficiency when compared to maceration. The operating conditions are typically room temperature and atmospheric pressure, but heating can also be used. Finally, compared to maceration and percolation, reflux extraction has a higher efficiency, despite the fact that the time of extraction and the amount of solvent required are slightly lower. Moreover, the operational parameters are atmospheric pressure and heating [13].

New extraction techniques are characterized by shorter extraction times, lower operational temperatures, reduced solvent amount and process automation. Moreover, because of the previously mentioned benefits, these techniques are regarded as environmentally friendly. Different parameters should be optimized to obtain higher yields of the target analyte. Thus, in these new methodologies, common parameters to be optimized include solvent ratio, extraction solvent, extraction time, pH, temperature and particle size [16,17]. New methodologies for extracting seaweed compounds include ultrasound-assisted extraction (UAE), supercritical fluid extraction (SFE), microwave-assisted extraction (MAE), enzyme-assisted extraction (EAE) and pressurized liquid extraction (PLE).

UAE is based on the application of low-frequency (16–100 kHz) and high-power (8–20 W) waves to disrupt cells, releasing target analytes, accelerating the diffusion and increasing mass transfer [17]. This technique has been used successfully to extract pigments, phenolic compounds and carbohydrates from different seaweed species [18]. SFE is based on the use of solvents at pressures and temperatures above their critical points, so solvents are denser than gases but have comparable viscosity and intermediate diffusivities to liquids and gases. This method has been used to extract carotenoids, chlorophylls, PUFAs and polyphenols [17]. MAE is based on the heat produced by the direct interaction of electromagnetic waves (usually 2.45 GHz) with polar solvent molecules through dipole rotation and ionic conduction [17]. MAE has been used to extract several seaweed compounds, including carbohydrates and proteins [19]. EAE is a very selective and specific method since enzymes are used to degrade the cell wall of algae cells. For this process, the enzyme concentration and the optimal enzyme reaction conditions should be optimized to improve specificity and selectivity [17]. This extraction method has been used to extract phlorotannin, proteins or hydrocolloids from various seaweeds [20].

PLE is a green extraction technique that involves extracting analytes from a solid matrix using high temperature and pressure, typically between 50–20 °C and 3.5–20 MPa [21]. With these conditions, both solubility and mass transfer rates are increased, leading to a solvent diffusivity increment and, therefore, meliorating matrix kinetics [17]. In this way, an experimental design is needed so all the parameters can be selected to guarantee optimal operational conditions [22]. Moreover, PLE allows the use of several solvents, including green extraction solvents such as water, and a mixture of water with ionic liquids or eutectic solvents. When water is used as the solvent, this technique is also known as high-pressure solvent extraction (HPSE), accelerated solvent extraction (ASE), enhanced solvent extraction, pressurized fluid extraction (PFE), pressurized hot solvent extraction (PHSE) or subcritical water extraction (SWE) [23]. When compared to the SFE technique, PLE operates without reaching the critical point of the liquid solvent and allows the use of broader range of solvents [21,22]. Moreover, PLE requires less extraction time than other traditional extraction techniques, such as Soxhlet extraction. In fact, the extraction time ranges from 5 to 20 min [24]. Furthermore, the use of PLE allows the achievement of higher yields, despite the fact that is not suitable for the extraction of thermolabile compounds and is not as selective as SFE [21,25]. However, PLE may be considered as a suitable green extraction technique to extract different bioactive compounds including polysaccharides, proteins and PUFAs since non-toxic solvents and high extraction yields are obtained. Considering the non-toxic solvents used, the extraction of these compounds with PLE may be an interesting pathway for the food industry.

The aim of this study was to compile, analyze and organize the available data on seaweeds components and the use of PLE as a potential extraction technique to obtain active compounds of interest to the food industry. Furthermore, the effects of combining PLE with other novel techniques for increasing extraction yield were revised. Finally, the use of different compounds extracted from edible seaweeds in the food industry was summarized to identify a potential new pathway in this production sector.

## 2. Matrix Components

This section compiles the main characteristics and interest of different compounds found in seaweeds. It is important to highlight that the information described is primarily aimed at the food industry, although pharmaceutical approximation was also considered. Moreover, the nutritional composition of different microalgae is compiled in Table 1 to provide comprehensive information.

### 2.1. Proteins

**Figure 1:**
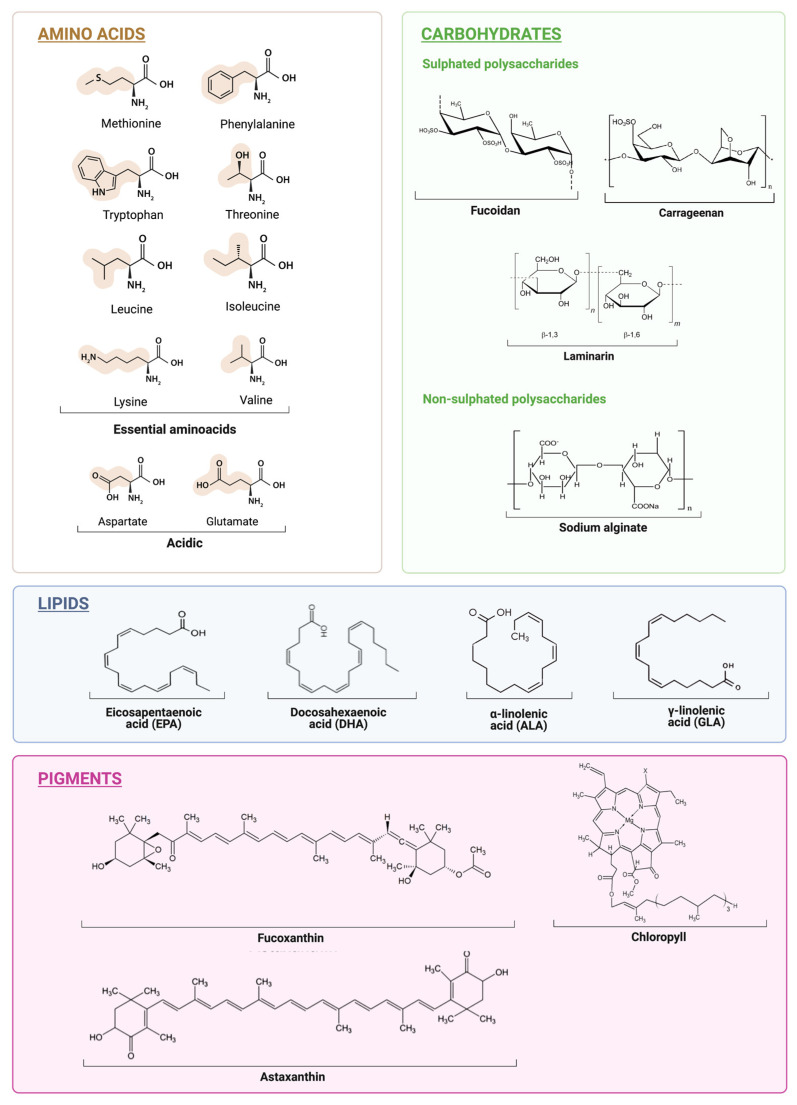
Chemical structure of the main amino acids, polysaccharides, lipids, and pigments extracted from edible seaweeds.

Proteins are large molecules composed of smaller units known as amino acids that are linked together with aminoacidic bonds. Considering the amino acid profile of seaweeds, it is important to note that most of them contain all the essential amino acids, with aspartic and glutamic acid being particularly abundant. Figure 1 depicts the chemical structure of these amino acids found in edible seaweeds. *Ulva lactuca* (a green edible seaweed) has an amino acid profile that is like that recommended by the Food and Agricultural Organization (FAO) and the World Health Organization (WHO). Seaweed proteins are suitable for inclusion in food formulations due to their amino acid profile and agglutination properties [4]. Several authors have proposed producing antioxidant hydrolysates by hydrolyzing seaweed proteins. Researchers used commercial enzymes to hydrolyze *Ecklonia cava*, *Ishige okamurae*, *Sargassum fullvelum*, *Sargassum horneri*, *Sargassum corearum*, *Sargassum thunbergii* and *Pyropia columbina* to obtain bioactive peptides. *P. columbina* increased its antioxidant activity after a simulated gastrointestinal digestion [34]. Thus, since some studies have found drawbacks in the digestibility of proteins from some seaweeds, the use of cellulases, xylanases and β-glucanases has been studied to improve the digestibility of protein from *Palmaria palmata* [15]. Furthermore, seaweed proteins such as phycobiliproteins have been linked with anti-inflammatory, hepatoprotective and antioxidant activities [46].

**Figure 2:**
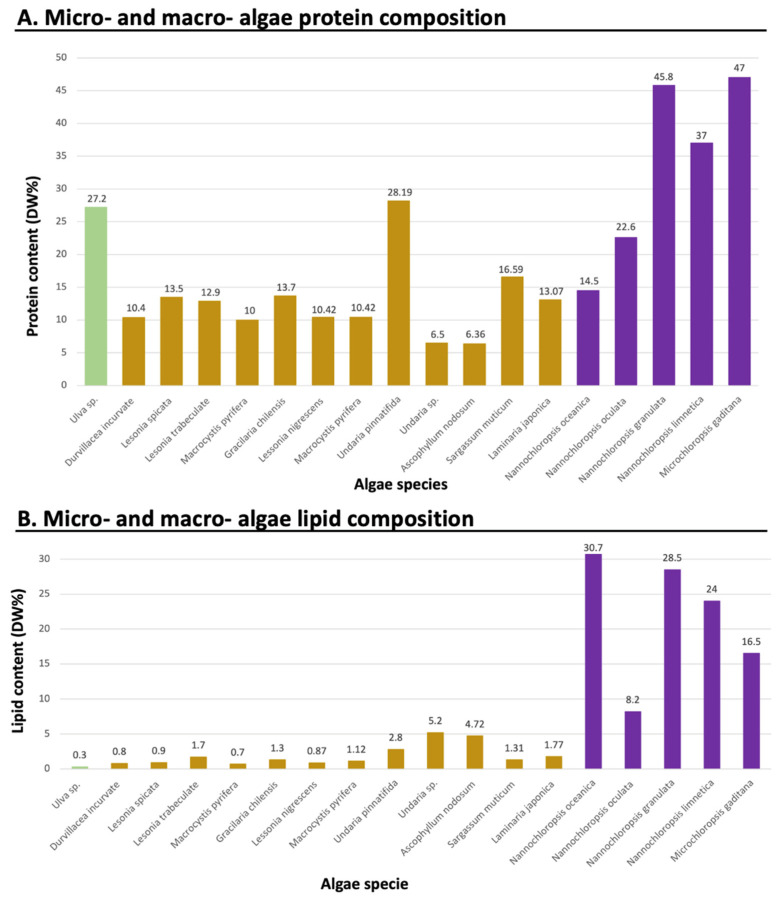
Graphical comparison between different microalgae and macroalgae protein (**A**) and lipid (**B**) composition, expressed as dried weight (%). Green color refers to green seaweeds, brown color refers to brown seaweeds and purple color refers to microalgae species.

Moreover, a relationship has been discovered between seaweed bioactive peptides and metabolic syndrome. The metabolic syndrome is a collection of medical conditions that can lead to different cardiovascular diseases, and it can be avoided with functional foods. Indeed, NMEKGSSSVVSSRMKQ is the first antithrombotic peptide produced by hydrolyzing *Porphyra yezoensis* proteins with pepsin. This peptide binds to the coagulation pathway and inhibits it. Furthermore, seaweed-derived bioactive peptides inhibit some enzymes involved in the renin–angiotensin–aldosterone system (RAAS), which plays a key role in the hypertension treatment [4]. Microalgae proteins have been reported to be good gel and foam formers, with *Arthosphira platensis* being particularly notable. Moreover, microalgae are also an interesting source of proteins and bioactive peptides, as shown in Table 1. A study published in 2022 found that the protein composition of *Nannochloropsis granulata* and *Microchloropsis gaditana* was high, with 45.8 and 47% expressed as DW, respectively. Furthermore, studies revealed that microalgae peptides from different genera, including *Chlorella, Nitzschia* and *Bellerochae* are distinguished by their antihypertensive, antibiotic and antiviral activities [40,41]. Figure 2 depicts a graphical representation of different micro and macroalgae protein composition, except for *Nannochloropsis granulata, Nannochloropsis limnetica* and *Microchloropsis gaditana*, which had higher protein content. Based on the data presented, seaweeds are a suitable and complete source of proteins that can be incorporated into a variety food products.

### 2.2. Carbohydrates

Seaweed contains monosaccharides, disaccharides and polysaccharides. Polysaccharides found in seaweeds are classified into two groups: sulphated and non-sulphated. As is shown in Figure 1, sulphated polysaccharides include fucoidans, carrageenan and laminarin, while non-sulphated polysaccharides are mainly alginates [47]. Seaweed polysaccharides and fiber are not digestible by humans. Moreover, in vitro studies have shown that polysaccharides from *Undaria pinnatifida*, *Laminaria japonica* and *Hizkia fusiformis* inhibit pepsin activity by 21%, 55% and 41%, respectively [15]. Despite their inability to be digested, these compounds serve as prebiotics in the human body because they can be degraded by intestinal bacteria [3]. However, it is important to highlight that some studies have linked prebiotic consumption to human flatulence. In fact, 14 women were studied for 4 weeks after consuming inulin, and 12% of the volunteers experienced severe flatulence [48]. Moreover, because seaweeds are composed of soluble fiber with high capacity to retain water (CRW), they can be used as hydrocolloids in food formulations. Some of the functions of hydrocolloids include thickening, stabilizing and emulsifying [3].

Carrageenan is a sulphated linear galactant found in 71% and 88% of *Chondrus crispus* and *Kappaphycus* spp., respectively. Carrageenan gel form from *Chondrus crispus* has been shown to have antiviral and anticoagulant properties against HIV and herpes simplex virus (HSV). The anticoagulant capacity of this compound has been related to the sulphate molecules in the polysaccharide chains [4].

Alginates are non-sulphated linear unbranched polysaccharides found in the intercellular spaces of brown algae [4,49]. *Laminaria*, *Saccharina*, *Lessonia*, *Macrocystis*, *Durvillaea*, *Eckonia* and *Ascophyllum* are the main seaweeds used to obtain this polysaccharide. Alginates are used in the food industry due to their technological properties, as these compounds are characterized by their gelation ability and become insoluble as a result of the formation of a cross-linked structure. Because of this, alginate is an excellent material for the active edible coating systems on foods such as fruits and vegetables [50]. Furthermore, the Food and Drug Administration (FDA) designated alginic acid and its salts as generally regarded as safe (GRAS) ingredients for oral administration [4].

Laminarins (Figure 1) are the main storage polysaccharides found in the cytoplasm of brown algae [40]. This small glucan is isolated from brown seaweeds and has a molecular weight that ranges from 1 to 10 kDa [51]. Laminarin can be found in a variety of seaweed species, including *Laminaria* spp., *Ascophyllum*, *Fucus* and *Undaria* [4]. This compound is well known for its gelling and emulsifying properties and is commonly used in the food industry as an additive [51]. Moreover, several studies have shown that laminarin has anti-apoptotic, anti-inflammatory, immunoregulatory, antitumor, anticoagulant and antioxidant activities [52]. These biomedical properties may be related to the sulphated composition of seaweed polysaccharides, which are not found in the terrestrial plants [4]. When the average molecular weight of laminarin was reduced to six kDa, its antioxidant activity increased from 7.5 to 79.7%. This could be explained by the fact that as the average molecular weight decreases, the number of carbonyl groups increases, interacting with transition metal ions and enhancing lipid oxidation protection [51]. Moreover, laminarin has a positive effect on the biochemistry and microbiology of the human gut microflora, modulating the intestinal metabolism in a positive way [4].

Fucoidans are sulphated polysaccharides (SP) found in brown seaweeds such as *Saccharina japonica*, *Laminaria ochroleuca* and *Himanthalia elongata*. The anti-inflammatory, anticoagulant, antitumoral, anti-thrombotic, antioxidant and antiviral activities of fucoidans have been associated with their sulphation level [34,40]. An in vitro and in vivo study of *Fucus evanescens* fucoidan’s anticoagulant activity revealed that it has similar anticoagulant properties to heparin [4]. The anticoagulant activity has been related to the sulphation level, carbohydrate content and the position of sulphated groups on sugar residues [53]. In addition, fucoidans may alter the cellular surface properties effectively preventing virus penetration, and as a result, antiviral activity [53]. A study using *Laminaria japonica* as a source of fucoidans showed its ability to scavenge superoxide radicals and hypochlorous acid. The low molecular fraction of *L. japonica* fucoidans also had a significant inhibitory effect on low-density lipid (LDL) oxidation induced by Cu^2+^ [53]. Thus, fucoidans may be used to prevent free radical-mediated diseases. Finally, the antitumor activity of fucoidans from brown seaweed was studied, and it was discovered that this sulphated polysaccharide could inhibit the proliferation of tumor cells by stimulating the apoptosis, blocking tumor cell metastasis, and enhancing immune response. These antitumor properties may lead to the use of fucoidans as functional ingredients or nutraceuticals [53]. Considering all the data presented and the increased interest of consumers in bioactive compounds in recent years, fucoidans may be considered as functional ingredients in the food industry [53].

The composition of microalgae polysaccharide (Table 1) has also been studied, showing immunomodulatory activity, low blood sugar and lipid levels in vitro and prebiotic activity [27,28]. Moreover, sulphated polysaccharides derived from *Arthosphira*, *Chlorella*, *Phaeodactylum*, *Schizochytrium* and *Thrautochytrium* showed antiviral, antitumor, and anti-inflammatory activity [29,30].

### 2.3. Lipids

The lipidic fraction in seaweeds varies between 1–8%, with the most common long-chain polyunsaturated fatty acids (PUFAs) found in seaweeds being γ-linolenic, α-linolenic, eicosapentaenoic and docosahexaenoic acid, whose chemical structure is shown in Figure 1. Because PUFAs have been linked to the prevention of cardiovascular, diabetes and hypertension diseases, their presence in seaweed has an interesting functional activity [3]. Moreover, the w-6/w-3 ratio is commonly used to define lipid quality [7]. According to the data, the w-6/w-3 ratio is 15/1 in Western diets, while the FAO recommends a ratio of less than 10. Because a high w-6/w-3 ratio is associated with the progression of various coronary diseases, the WHO recommends substituting saturated fatty acids for polyunsaturated fatty acids [6]. Furthermore, the atherogenic index (AI) and thrombotic index (TI) are parameters that indicate the lipid deposition in the artery wall as well as the thrombotic effect of saturated fatty acids (SFAs), respectively. Both indices are widely used to assess the quality of SFAs. In 2019, a study on the bioactive fatty acids extracted from *Laminaria ochroleuca* was conducted, with AI and TI results comparable to those obtained from some fish species [7]. It is important to note that the fatty acids in seaweeds are in a phospholipid and glycolipid form, which confer the cell wall membrane. This allows for very little degradation during digestion. Hence, a mechanical disruption of the cell wall is needed in order for the seaweed lipid content to be released and absorbed [3]. To demonstrate the differences between microalgae and macroalgae species, the lipid composition of each was compared. As is shown in Figure 2, the lipid composition of microalgae is richer than that of seaweed. In fact, the lipid composition of microalgae varies depending on the genus (Table 1), reaching 46.12% of DW in some species of *Nannochloropsis*. Moreover, the lipid composition of microalgae is rich in PUFAs such as EPA, ARA and DHA, which has been linked to different biological properties such as anti-inflammatory, anti-diabetes, antithrombotic and anticancer activities, as well as a high capacity to prevent cardiovascular diseases [31,32,33,38]. Although the lipid content of microalgae is higher than that of seaweed, based on the data presented, seaweed may be a valuable source of quality fatty acids for the food industry, capable of being used in vegetarian and vegan formulations.

### 2.4. Pigments

Chlorophyll (Figure 1) is one of the most abundant pigments on the planet, and it has mainly been studied in higher plants [54]. The significance of this phytochemical is due to its antimutagenic and antigenotoxic activity [54] as well as its potential use in the food industry as a natural pigment. A study conducted in 2017 identified the chlorophyll profile, distinguishing over 31 pigments. Although extraction protocols for these pigments in seaweeds are not well developed [55], the abundance of these phytochemicals in edible seaweeds makes this matrix a good source of them. Moreover, the stability of chlorophyll in fresh and cooked *Porphyra* seaweeds was studied during in vitro digestion. The bioaccessibility of cooked *Laminaria* chlorophyll seaweed improved, while processing decreased this parameter [54].

Fucoxanthin (FUCO) is a carotenoid found in the glycoglycerolipids of brown algae chloroplasts and is involved in the photosynthesis process [8,56]. FUCO accounts for 10% of total carotenoids in nature and has a market with a 2.47% annual growth rate. This pigment is present in several algae genera, with *Undaria pinnatifida* having the highest concentration. The main bioactive properties of FUCO are the antioxidant, anticancer and anti-inflammatory activity, as well as the cytoprotective and skin protective effects [8]. The antioxidant effect of this compound is explained by the presence of an allenic bond and an acetyl functional group in its structure (Figure 1), both of which can scavenge different free radicals. Thus, it has been demonstrated that FUCO reduces the production of intracellular ROS and DNA damage while increasing glutathione levels, which is a key molecule in oxidant defense and the maintenance of the redox cell homeostasis. All these actions contribute to the prevention of apoptotic processes [8]. The skin protective effect of FUCO has also been studied using oral administration. Results showed that this compound suppresses transcription of the melanogenesis factor by inhibiting mRNA expression. Therefore, FUCO could be used to prevent harmful effects of ultraviolet (UV) radiation, such as melanomas [8]. The ideal conditions for FUCO incorporation into food formulations have already been studied, with the conclusion that an encapsulation should be made with a solution with pH 5–7 and it should be stored at 4 °C [8]. 

### 2.5. Metals

As previously stated, seaweeds are a rich source of bioactive compounds that can be used in the food industry. However, seaweeds also contain significant amounts of metals. In fact, seaweed consumption has been considered as a high-risk route for heavy metals and metalloids due to their high capacity to bioaccumulate these compounds. Thus, the main metals found in these matrices are lead (Pb), cadmium (Cd), mercury (Hg), and arsenic (As) [57]. Red algae are high in selenium (Se), manganese (Mn), nickel (Ni) and silver (Ag), while brown seaweeds are high in copper (Cu), cobalt (Co), chromium (Cr), As and iron (Fe). Finally, zinc and Pb are commonly found in green seaweeds [58]. These metals are toxic and persistent, and their consumption may cause endocrine disruption and carcinogenic activity [59]. However, the potential risk of consuming seaweeds grown in Saint Martin’s Island was studied, which is potentially a risk zone for heavy metal accumulation. A total of 21 heavy metals and metalloids were analyzed, and no health risk was found because bioaccumulation was below the established limits (Hazard Quantities < 1) [57]. Another study analyzed 11 species of seaweeds grown in South China Sea, showing a high degree of variability and complexity [58]. In this way, considering the importance of ensuring the consumers’ health, heavy metal and metalloid analysis of the species used is required.

## 3. Pressurized Liquid Extraction (PLE)

### 3.1. General Aspects of PLE

PLE is an extraction technique that consists of the removal of analytes present in a solid matrix by applying high temperatures (*T_extr_*) and pressure (*P_extr_*), usually up to 200 °C and over 200 bar, respectively according to Nieto et al. [22], without reaching the critical point using liquid solvents [23]. These conditions increase solubility and mass transfer rates, resulting in increased solvent diffusivity and, as a result, improved matrix kinetics [17].

Temperature, pressure, time, number of cycles, sample weight and solvent all influence extraction yield. To improve the efficacy of PLE, these parameters should be optimized by using a proper experimental design [22].

**Figure 3:**
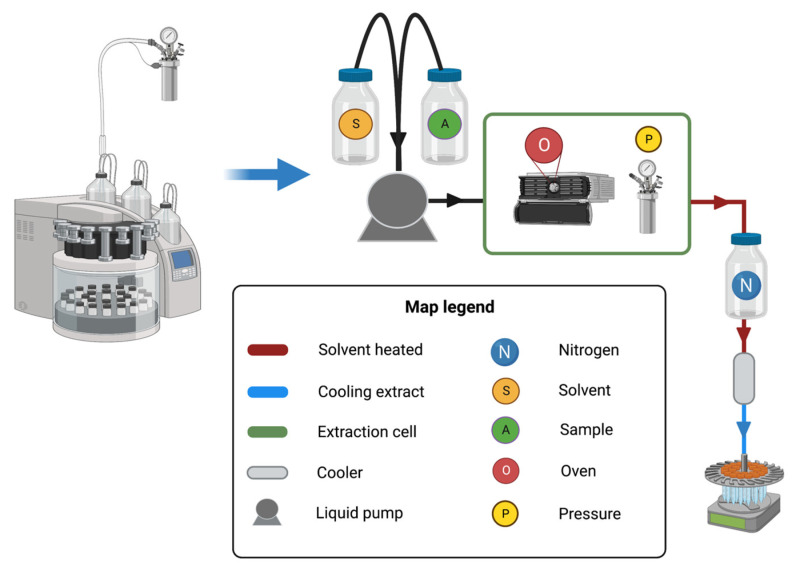
Pressurized liquid extraction equipment and schematic representation of its operation. First, the solvent (S) and the sample (A) are injected into the extraction cell. The extraction cell is composed of an oven (O) and a pressure valve (P) which together allow the achievement of the temperature and pressure selected to extract the compound present in the sample. Then, the extracted compound is cooled and collected in a carousel.

Figure 3 depicts a schematic representation of the PLE extraction equipment’s operation. A high-pressure pump feeds the solvent into an extraction cell and the *P_extr_* in the system is kept constant [24,60]. Because operational *T_extr_* and *P_extr_* control is critical in this method, the extraction cell is kept in an oven with different valves and restrictors [60]. Moreover, an extract cooler system, a back pressure regulator and a vial to collect the extract are required [24]. Finally, it is important to keep in mind that the equipment must be resistant to corrosion and high pressure [24].

PLE can be used in three modes of action: static mode, dynamic mode and a combination of the two. The static mode is characterized by the use of constant temperature and pressure values, resulting in the sample being in contact with the solvent for a set period. On the contrary, in the dynamic mode, the solvent (usually water) flows continuously through the sample. As a result of the higher volume of the extract obtained, the analytes are diluted in the liquid extract. Analytes are typically pre-concentrated by liquid–liquid extraction or by solid-phase extraction to address this issue. Finally, a combination of both modes of action can be used, which may improve analyte extraction [23].

### 3.2. Sample Pre-Treatments

**Figure 4:**
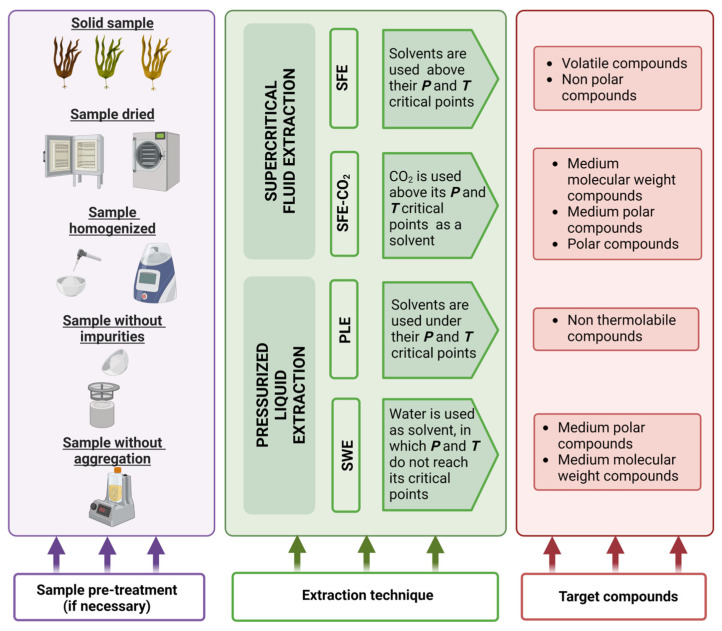
Pre-treatment steps, extraction techniques and target compounds from seaweeds using PLE and SWE. In purple, a schematic representation of the steps that should be followed to prepare the sample before the extraction technique is applied. In green, PLE and SPE operational conditions considering water and CO_2_ as solvents, respectively. In red, a comparison between the compounds extracted using each extraction technique.

Before using PLE, samples must be pre-treated to increase the contact surface between the solvent and the matrix during the extraction [23]. Pre-treatment can be compiled into four steps, as explained below and in Figure 4:

− Drying: the objective of this step is to remove water from the matrix, increasing extraction efficiency [23]. Air-drying, oven heating or lyophilization can all be used, with the latter being the most advantageous because it takes less time and does not degrade the compounds. Indeed, when non-polar solvents are used, this step is critical and a desiccant is commonly included in the PLE cell [22].
− Homogenization: by grinding, the sample should be distributed in a homogeneous manner [22].
− Sieving: this step increases the surface area of the analyte as well as the diffusion of the analyte from the matrix to the solvent [23]. This step yielded a similar particle size in which 2 mm is commonly used for PLE [22]. After sieving you can carry out grinding of the separated portion with greater particle size.
− Dispersion with an inert material: this step is recommended for some samples to avoid aggregation of particles that may lead to alteration in the extraction efficiency [23].

### 3.3. Relevant Parameters in PLE

#### 3.3.1. Solvent

One of the most important parameters to optimize is the extraction solvent [22]. The solvent’s function is to solubilize the target analytes while minimizing the extraction of other components [23]. Therefore, it is important to choose a solvent that has the same polar behavior of the target analytes [22]. Non-polar and water immiscible solvents or a combination of non-polar with medium-polarity solvents are used for non-polar or lipophilic compound extraction. Consequently, solvents with high polarity are used to extract polar and hydrophilic compounds. Finally, when extracting analytes with different polarities, a mixture of solvents with high and low polarity is commonly used. Indeed, some authors suggest following two PLE extractions when the target is for high and low polar analytes, so they can be removed in two steps [23]. As a result, because affinity and miscibility are the two parameters used in predictive approaches to determine the solubility of the target compound in green solvents at different temperatures, experimental trials may be limited [24].

Regarding the application of PLE on seaweeds, using water as a solvent is the most common green technique applied for the carbohydrates extraction since they are more soluble in water at 100–150 °C and the dielectric constant of water is reduced at this temperature. Moreover, subcritical water acts as an acid or an alkali, helping the polysaccharides extraction [47]. A study conducted in 2022 in which PLE was optimized by varying different parameters, showed that temperature was the most critical for the extraction of carbohydrates in microalgae, which can be extrapolated to seaweeds. Furthermore, bioactive polysaccharides extracted from seaweeds using PLE with water are not degraded because temperatures are kept below 200 °C, avoiding caramelization and other degradation reactions [47]. Although water is a good green solvent, it is important to consider that its use may result in unwanted reactions or interference coextraction, affecting the procedure’s selectivity [17]. Therefore, other eco-friendly solvent alternatives, such as deep eutectic solvents, are being considered for carbohydrate extraction. Deep eutectic solvents (DES) are eutectic mixtures composed of hydrogen bonding acceptors (HBAs) and hydrogen bonding donors (HBDs) [61]. Due to their stability, cost-competitiveness, and ease of synthesis, DES have been proposed to dissolve different polysaccharides such as cellulose, starch, chitin and lignin for biomass processing. Moreover, DES was recently used as a solvent to extract fucoidans and alginates from brown algae. The results of this study showed that DES functioned as a catalyst, yielding twice as much as acidified water extraction. Table 2 summarizes the information explained above by showing different solvents used in PLE extraction, and their target analytes.

When selecting a solvent, it is also important to consider subsequent steps of the process, such as the clean-up step or concentration step if necessary. Selectivity is the parameter that determines whether or not purification and concentration are required, and it is critical when developing a green technique process [69]. Finally, the solvent used must be both physically and chemically stable. Water, ethanol, organic esters such as ethyl acetate and ethyl lactate, (+)-limonene and their mixtures are the most commonly used solvents in PLE [24].

The operational conditions used to extract bioactive compounds from seaweeds using PLE are shown in Table 3. In 2017, for example, a study on the accuracy of some green solvents with PLE for the fucoxanthin extraction was conducted. Limonene, ethyl lactate and ethyl acetate were selected as green solvents and their ability to extract fucoxanthin was compared to that of ethanol [69,70]. The highest yields were obtained for each solvent when the operating temperature was set to 100 °C. None of the green solvents reached ethanol’s yield, with ethyl lactate had the highest percentage. Despite the yield results, limonene had the highest selectivity (expressed as the ratio of total carotenoids to total chlorophylls), proving that limonene is a good alternative green solvent for fucoxanthin extraction.

#### 3.3.2. Temperature and Pressure

As previously stated, *T_extr_* and *P_extr_* are two important parameters to be optimized when using PLE. Elevated temperatures are used to reduce the viscosity of the liquid solvent used, allowing it to a better wet the matrix, and solubilize the analytes of interest. In addition, diffusion of analytes in the matrix surface is facilitated because high temperatures aid in the breakdown of the analyte–matrix bonds [23]. *T_extr_* varies between 50 and 200 °C and is dependent on the target analyte. Thus, lower *T_extr_* are selected for extraction of certain bioactive compounds due to their thermolability. Because high temperatures above the atmospheric boiling point are required in order to keep the solvent liquid, a high operational *P_extr_* is required [23]. Furthermore, high pressure increases the extraction yield by forcing the solvent to enter the matrix pores [60]. In PLE methodology, *P_extr_* usually varies from 5.0 to 15 MPa. These high-pressure and temperature conditions allow for the extraction of the target analytes in a short period of time while using less solvent and showing a recovering ability in terms of extraction yield similar to other techniques [22].

#### 3.3.3. Time and Number of Cycles

The time of extraction is defined as the duration of direct solvent contact with the sample for a given *T_extr_* and *P_extr_* [24]. This value is determined by a variety of factors, including the mode of action. When using static mode d, extraction time is reduced (*t_extr_* = 5–20 min) [24]. On the contrary, when the dynamic mode is established, the flow of the solvent must be determined to select *t_extr_*. Furthermore, it is significant to notice that low flows cause PLE system blockages while high flows result in diluted extracts. Finally, it is known that several cycles with low volume lead to higher yields of the target analyte, while a single extraction with a large amount of solvent does not correspond with higher extraction yields [24].

### 3.4. Post-Extraction Treatment (Clean-Up)

During PLE, some compounds of the matrix could be co-extracted causing interferences, so a clean-up step could be necessary to decrease the limit of detection (LOD) value [22]. Extraction and clean-up steps can be carried out simultaneously, which leads to a reduction in time and quantity of solvents used, between 15% and 52% [78]. Different clean-up techniques can be used:

− On-cell clean-up technique: the solvent is passed through the cell to elute interferences prior to the extraction step. This reduces the extraction time required and enables the process to be automated. On the other hand, the analyte must have a different polarity than the compounds that cause the interference. Moreover, finding a solvent capable of eliminating the interferences without causing damage to the analytes can be difficult [78].
− Liquid chromatography techniques: they are used to remove interferences from complex matrices. The most used techniques are normal phase liquid extraction (NPLE) and gel permeation chromatography (GPC). On the one hand, NPLE is used as a preparative chromatography in a glass column filled in-house with the stationary phase [78], whereas GPC is a purifying technique based in the separation depending on the molecular size of the compounds. The main advantages of GPC are its ability to be automated and the clean-up capacity maintenance for months. Divinylbenzene-linked polystyrene gel is the main material used for GPC [22].

## 4. Combinatorial Approaches of PLE with New Extraction Methodologies

**Figure 5:**
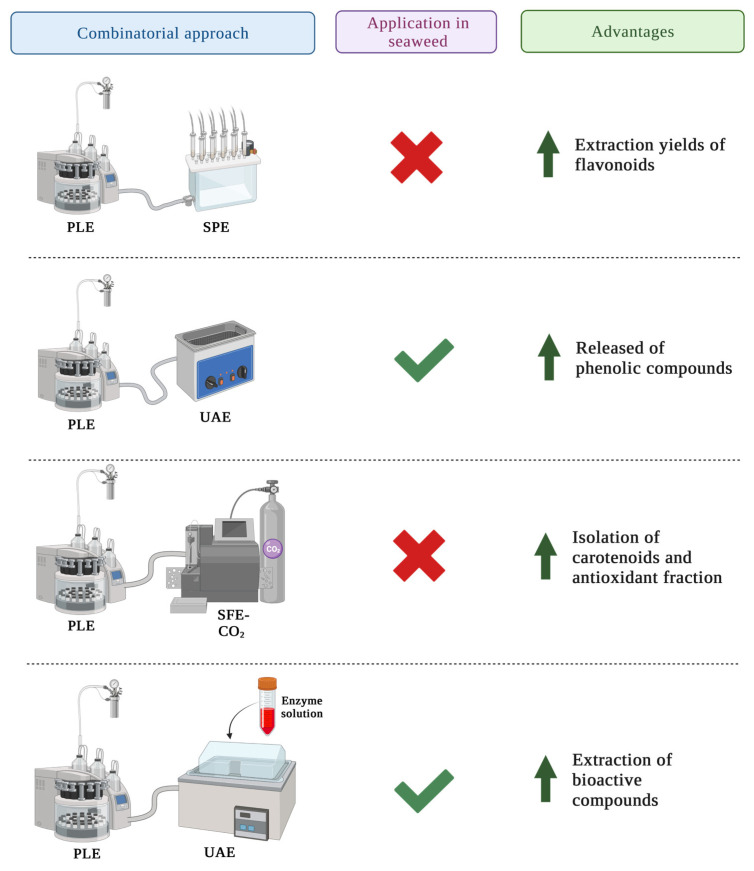
Improvements achieved in the recovery of compounds from different matrices when PLE is combined with other extraction techniques.

As shown in Table 3, there have been few studies on the use of PLE in seaweeds due to its novelty. However, following the green technology trends, PLE could be combined with other methodologies to improve bioactive compound extraction efficiency and reduce solvent and time consumption. Moreover, because combinatorial approaches of PLE applied in the extraction of bioactive compounds of seaweeds have not been thoroughly studied, results of trials where PLE and other techniques are applied in other matrices are shown in this section and compiled in Figure 5 as case studies for future applications of these techniques combined.

### 4.1. PLE Combined with SPE

The combination of PLE and SPE has been used for the separation of specific phenolic compounds [79]. The mode of action of this combination is based on the ability of PLE to extract bioactive compounds from the matrix and the ability of SPE to purify the extracted compounds [80]. Thus, SPE is mainly used as a post-extraction technique since PLE is a non-selective methodology.

There are no data on the use of PLE and SPE for the extraction of phenolic compounds from seaweeds, but it was applied in other matrices such as apple pomace, mate leaves and lemon peel. Higher yields of total flavonoids were obtained in all the three matrices, when compared to the extraction using PLE alone [79,80,81]. On the contrary, when lemon peel was used as the matrix, the yields of the polar compounds were lower, while total phenolic acids and flavonoids showed no statistical difference between PLE and PLE combined with SPE in mate leaves [80,81].

### 4.2. PLE Combined with UAE

Some studies have combined the use of PLE with UAE (UAPLE) in different matrices, including seaweeds. A recent study combined PLE and UAE to extract phenolic compounds from three brown and one red algae. The operational conditions (solvent 80% MeOH:H_2_O (*v*/*v*); 10 mL; 130 °C; 130 bar; two static cycles of 10 min) were able to increase the release of phenolic compounds from the matrix due to the high and stable pressure [82]. Nevertheless, further research is needed to know if scale-up is available from the economic point of view [83], since many companies have difficulties because of the high expense with facilities, extraction time and ultrasound power [84].

### 4.3. PLE Combined with SFE-CO_2_

The combination of PLE with SFE-CO_2_ is a sequential process based on the ability of SFE-CO_2_ for the extraction of the lipophilic fraction of the matrix, and the ability of PLE for the extraction of the antioxidant or high polarity compounds [85]. Because there is no information available about this sequential process used in seaweeds, results from other studies were compared to determine if this methodology could be useful for extracting seaweed compounds. For the recovery of bioactive compounds from rowanberry pomace using SPE-CO_2_ and PLE consecutively, results showed that this is an effective method for the isolation of carotenoid-rich and antioxidant-rich fractions [86]. Same conclusions were achieved when this sequential process was applied in *Viburnum opulus* pomace and berries [85]. Moreover, an economic evaluation of this process applied in passion fruit by-products was carried out in Brazil. This study showed that the combination of these techniques is economically applicable in large-scale production since it increases process productivity and decreases the cost of manufacturing [87].

### 4.4. PLE Combined with EAE

The application of EAE as an extraction technique leads to some disadvantages, such as the high cost of enzymes, the limitation of cell disruption because of the specificity of the enzyme and the inactivation of enzymes with parameters such as temperature and pH change [88]. To solve these limitations, EAE studies combined with other new methodologies have been performed. For example, when EAE was combined with alkaline hydrolysis and PLE for the extraction of bioactive compounds from *Sargassum muticum*, the extraction yields were higher than when PLE was used alone [89]. This could be explained by the formation of a protein–polyphenol complex which results in decreased polyphenol recovery. This complex may be formed when the enzyme disrupts the cell of the seaweed, releasing proteins and other compounds. Thus, these compounds may form complexes with polyphenols, resulting in aggregation and precipitation and ultimately, lower yields [89]. Because no additional information was discovered, more research is required to determine whether combining EAE and SPE could result in higher yields.

Given the current data on PLE combined with various new methods, a sequential process using PLE and SFE-CO_2_ should be considered because extraction yields are increasing, and an industrial scale appears to be feasible. However, because there has been no research on the application of a sequential process involving PLE in seaweeds and different parameters affecting the percentage of recovery, including the matrix, more research is required. Furthermore, because UAPLE produces intriguing results, scale-up trials should be conducted to determine whether this sequential process is economically feasible.

## 5. Evaluation of Pressurized Liquid Extraction (PLE) Applications

As mentioned in previous sections, using PLE to recover seaweed compounds results in the extraction of various bioactive compounds. Because of their technological function, importance as functional ingredients, or application in innovative food packaging systems, these compounds can be used in the food industry.

### 5.1. Technological Function and Functional Ingredients

Different compounds extracted from seaweeds are already used in the food industry for the technological improvement of food products, such as carbohydrates from seaweeds which are mainly used for its functional properties. Thus, agar is applied in the confectionary industry for its hydration maintenance capacity. Moreover, the addition of agar in meat products allows the reduction of the fat content in the final product [90]. The use of seaweed extracts in meat emulsions is interesting from a technological point of view since a harder and chewier structure with better water and fat binding is achieved [91]. For example, due to the increase in vegan and vegetarian diets, meat analogues are increasingly in demand and carrageenan is used because of their stability properties [90]: it is already used in low-fat sausages, beef burgers and beef patties as a thickener and stabilizer agent [92]. In addition, since algae have essential micronutrients such as Mg, K and Fe, and low Na content, the addition of these extracts into meat products may be a good opportunity to increase the nutritional value of these products [93]. Carrageenan and alginates have been added in bakery products such as bread, being able to reduce the moisture loss during storage and the dehydration rate of the crumb. Additionally, alginate was able to retard the hardening of the crumb [94]. Fruit jellies, donuts and cakes are also examples of products where agar is added [90]. At last, the use of alginates has an antimicrobial growth activity in the vegetable industry and is a good choice for encapsulation and delivery systems of probiotics, according to the bibliography [90].

On the other hand, proteins, peptides and amino acids are mainly used as stabilizers, thickener agents, protein replacements and gelling agents [95]. Peptides extracted from different seaweeds were incorporated in pasta products, showing superior pasta quality and antioxidant properties over the control [95]. Furthermore, the addition of *Palmaria palmata* hydrolysate in bread improved texture and sensory acceptability. In addition, several studies of peptides extracted from seaweeds have been carried out, and Wakame peptide jelly and Nori peptide S are two bioactive peptides included in some Japan foods due to their antihypertension activity [4]. Nutritional supplements are also considered in the scope of the food application of seaweed extracts. As seaweeds are a good source of proteins, not only because of the quantity but for the quality, the use of this extract as supplementation would be a good option for those athletes following a vegan diet [96]. Thus, there are already products on the market, such as Solaray, that contain extracts of *Rhodymenia palmata*, which helps in the maintenance of the immune system health [77].

The incorporation of phlorotannin into food formulations may be limited because of their astringency and bitter taste. Thus, these compounds were encapsulated into nanofibers made of sodium alginate and polyethylene oxide and successfully incorporated into chicken breasts. The chicken was stored, and thanks to the phlorotannin’s encapsulation, *Salmonella* growth was prevented, while the sensorial characteristics of the product were unaffected [97]. Moreover, the preservation ability against polyphenol oxidase activity and melanosis formation was proved during white shrimps’ ice storage when phlorotannin extracted from *S. tenerimum* were added. Furthermore, when shrimps were immersed in 5% phlorotannin solution, shelf life was extended by 4 days and higher scores on overall sensory acceptability when compared to control [97]. Finally, different studies proved that the addition of seaweeds to the food formulation of different products leads to a reduction in cooking loss and in an improvement in the texture, as it is shown in Table 4 [98].

Finally, due to the current trend of changing the soy- and animal-derived protein sources for animal feed, seaweed extracts have been also incorporated into these products [95]. According to one study, incorporating algae extracts into dairy cattle feed resulted in higher I and Se content [99]. In fact, it has been demonstrated that including seaweed extracts in animal feed is a good way to achieve the I daily intake for those people with I deficiency, since milk excretion meets the needs of this mineral [95]. The incorporation of red seaweed extracts to poultry feed was also studied. The results show that incorporating *Sarchodiotheca guadichaudii* and *Chondrus crispus* extracts into layer feed improves the growth of beneficial bacteria and reduces *Clostridium perifringens* proliferation in the gut, thus improving the safety of the products obtained. Moreover, the egg yolk and weight were increased by adding 1% of *Sarchodiotheca guadichaudii* into the feed without altering the color of the yolk and the shell thickness [100]. Extracts from seaweeds are also being studied for feeding fish, especially protein extracts, since they are the most expensive dietary requirement for fish and shellfish aquaculture. Moreover, seaweed is also a good source of highly unsaturated fatty acids. Considering the requirements for fish nutrition, studies suggest that seaweed extracts may be a good option for fish feed, allowing the substitution of animal meal by plant meal in these products [95,101].

**Table 4:** Application of seaweed extracts in food products.

Product	Seaweed	Form	Technological/Nutritional Function	Ref.
Pasta	*Undaria pinnatifida*	Powder	Fucoxanthin was not altered	[102]
Pasta	*Sargassum marginatum*	Powder	Lower cooking loss Better gluten network when 2.5% of seaweed was added	[103]
Beef patty	*Undaria pinnatifida*	Dried and ground	Lower cooking loss Better texture Higher quantities of mineral and fiber when 3% of seaweed was added	[104]
Chicken breast meat	*Undaria pinnatifida*	Carotenoid pigment Fucoxanthin	Red and yellow color were increased Inhibition of lipid peroxidation after cooking	[98]
Restructured poultry steak	*Hymanthalia elongata*	Powder	Lower cooking loss	[105]
Pork emulsion meat	*Hymanthalia elongata, Undaria pinnatifida, Porphyra umbilicalis*	Dried and ground	Higher antioxidant ability Omega-3 was increased Higher mineral profile Higher amino acid profile (*P. umbilicalis*)	[93]
Low-fat Frankfurters	*Hymanthalia elongata*	Dried and ground	Higher levels of w-3 Higher quantity of fiber	[106]
Cod	*Fucus vesiculosus*	Extract and subfractions of phlorotannin	Inhibition of lipid oxidation in fish model systems	[107]
Fish oil-enriched granola bar	*Fucus vesiculosus*	Phlorotannin extract	Inhibition of lipid oxidation	[108]
Canola oil stored under favorable oxidation conditions	*Fucus vesiculosus, Ascophyllum nodosum, Bifurcaria bifurcata*	Phenolic compounds	Reduction of lipid oxidation	[109]
Pork homogenates	*Laminaria digitata*	Fucoidan extract	Reduction of lipid oxidation Oxidation of myoglobin	[110]
Fish oil-enriched mayonnaise	*Fucus vesiculosus*	Phenolic compounds	Prevention of lipid oxidation	[111]
Fish oil-enriched milk	*Fucus vesiculosus*	Phlorotannin	Prevention of lipid oxidation	[112]
Cookies	*Fucus vesiculosus, Ascophyllum nodosum, Bifurcaria bifurcata*	Phenolic compounds	Antioxidant effect	[113]
Bread	*Sargassum fulvellum*	Powder	The shelf life is increased Less hardness and gumminess	[114]
Bread	*Kappaphycus alvarezii*	Powder	High dietary fiber content	[115]

Considering the advantages of using PLE alone or in combination with other green techniques to obtain previously exposed seaweed bioactive compounds, and how food products may improve with the addition of these biomolecules, the incorporation of seaweed extracts may be a good strategy to enhance their nutritional profile and the technological properties.

### 5.2. Application as Innovative Food Packaging

Some compounds extracted from seaweeds such as laminarin, phlorotannin, flavonoids, terpenes, lactones and proteins are active against bacteria and fungi cells and bacteria biofilm formation (which is more difficult to remove) [116]. The correct preservation of organoleptic characteristics, while avoiding microbial growth during the storage as well as the need to extend the shelf life of the products, are critical for the food industry because these are factors deeply involved with the increase in food waste. Therefore, the use of antimicrobial compounds extracted from seaweeds may be a good option for increasing the shelf life of food products.

Sensory analyses of different products with some of these antimicrobial compounds were carried out to identify their impact on different parameters such as flavor, taste, color and smell. The results showed that edible film made of chitosan and seaweed extracts from *H. longata* and *P. palmata* inhibit the growth of mesophilic and psychrophilic microorganisms by maintaining the pH and water activity without affecting the sensorial characteristics of fish burgers. On the contrary, the sensory evaluation of pork patties with fucoidan and laminarian extract in a ratio of 0.5 *w*/*w* proved an adverse impact on the product. To avoid a possible negative impact on the organoleptic characteristics of the products, adding the antimicrobial compounds onto the packaging instead of adding them directly in the product could be an option. In fact, considering that most of the spoilage and contamination of food occurs on its surface, adding these compounds in the packaging may extend the shelf life of the product without affecting its organoleptic characteristics [116].

In terms of packaging, seaweed derivatives were studied to determine their suitability for bioplastic production. Bioplastics are synthetic plastics derived from biodegradable sources and their main disadvantage is their hygroscopicity [117], which affects the mechanical and storage properties required for food packaging. Furthermore, edible coatings are thin membranes composed of GRAS such as polysaccharides, lipids and proteins. Moreover, edible coatings can act as carriers of different bioactive compounds useful in food preservation. Thus, these special coatings maintain firmness, inhibit microbial growth and prevent food weight loss during long-term storage [50]. Carrageenan is a polysaccharide that can be used as an additive combined with other compounds for bioplastic production since it has low water vapor permeability (WVP) [117]. A biodegradable film made of olive extract, glycerol and 1% of carrageenan (*w*/*v*) showed good mechanical and antimicrobial properties. Moreover, a bionanocomposite made of 10% of gelatin (*w*/*v*), 0.5% (*w*/*v*) of k-carrageenan and 1, 3 and 5% of nano-SiO_2_ showed a drop in the WVP from 100% to 68%. Finally, the bioplastic production using starch, glycerol and 5% of carrageenan showed that the addition of carrageenan enhanced the moisture resistance, brittleness and the tensile properties of the polymer. Thus, carrageenan can be used to produce edible food packaging [117].

Alginates are another type of polysaccharides that could be used in the formulation of biodegradable films because their main properties are tensile strength, elongation and WVP that are suitable for biodegradable packaging [117]. To determine how mechanical properties were affected, alginate biofilms were compared using hydrophilic and hydrophobic plasticizers. Tributyl citrate (TC) showed better results because TC and alginate secondary interactions improved mechanical resistance. Furthermore, it was proved that elongation at break can be increased by using hydrophilic plasticizers such as glycerol [117]. A study using alginate with aloe vera (AV) and garlic oil (GO) in different proportions to produce a brand-new edible coating was performed to determine their UV shielding, thermal and antimicrobial properties. The results showed that after 16 days of storage, tomatoes with the edible coating made of 33.3% alginate, 66.7% AV and 5% GO showed an 8% mass loss while tomatoes without edible coating showed a 47% of mass loss. In addition, tomatoes with 33.3% alginate, 66.7% AV and 5% GO as edible coating suffered less damaged in the UV light measurement and showed better elongation break properties. For the inhibition growth of *Staphylococcus aureus*, *Escherichia coli* and *Syncephalastrum racemosum*, better results were obtained when tomatoes were coated with 33.3% alginate, 66.7% AV and 5% GO. On the contrary, better tensile strength results were obtained in tomatoes with 33.3% alginate and 67.3% AV [50].

Based on the data presented, the incorporation of alginates, carrageenan, laminarin, phlorotannin, flavonoids, terpenes and proteins onto innovative food packaging is a viable option for this innovative pathway.

## 6. Advantages and Drawbacks of the Application of PLE as an Extraction Technique of the Bioactive Compounds from Seaweeds

Although PLE is a better option for extracting compounds than traditional extraction techniques, the main disadvantage of this methodology is the high cost of the equipment. This is mainly due to the high requirements in terms of temperature and pressure, as well as the fact that the equipment must be made from materials that can withstand these conditions while avoiding corrosion [24,118].

Despite the high cost of the equipment, PLE has many advantages. On the one hand, one of the primary benefits of using PLE is that it has a lower environmental impact than other conventional extraction methodologies such as maceration and Soxhlet. This improvement is primarily due to a reduction in extraction time and a lower amount of solvent required. As previously stated, high temperatures allow for a decrease in solvent viscosity, resulting in faster solubilization and diffusion of the target compound [17]. Generally, extraction takes about 15 min [118]. This reduces the amount of energy required in the extraction process, making this methodology greener than other conventional methods. Solvents, particularly organic solvents, have traditionally been a problem in conventional extraction methods from an environmental standpoint. This is due to their classification as Volatile Organic Compounds (VOCs). VOCs are organic pollutants that contribute to the photochemical smog formation in the troposphere and ozone depletion in the stratosphere as a source of radical sources [119]. Furthermore, given that the food industry accounts for 2% of total global solvent consumption [119], primarily for extraction processes, it is necessary to develop techniques that reduce the amount of solvent used. Moreover, biobased solvents are commonly used in this technique. These are defined as solvents derived from biomass and characterized for their biodegradability, lower VOC content and near-zero carbon balance. The use of these solvents is even more critical because it is known that a portion of the solvent used remains in food, food additive excipients and packaging [119]. In PLE, the mainly biobased solvents used are alcohol, ethanol, ethyl acetate, methyl lactate, ethyl lactate and D-limonene. Then, PLE is considered as one of the novel techniques in which solvent consumption is not only low, but it is also better in terms of biodegradability and toxicity.

## 7. Conclusions and Future Perspectives

Nowadays, the scientific community is increasingly interested in obtaining bioactive compounds from novel matrices using less aggressive environmental methodologies. As explained in this review, PLE is a green extraction technique that allows the separation of target active compounds such as polysaccharides, lipids, proteins and bioactive compounds using short time cycles and low quantities of solvents due to the high-pressure and temperature operating conditions. In this way, since edible seaweeds are becoming more important in the Western world due to both their nutritional profile and their technological properties, using PLE as an extraction technique appears to be a viable option, according to the bibliography. Moreover, data from the combination of PLE with other extraction techniques were evaluated to determine if they were appropriate, and satisfactory results were obtained. There are little data available on the combination of PLE with other extraction techniques using edible seaweeds, but comparable results are expected based on the results obtained with other matrices.

However, even though the data available today are primarily focused on the pharmaceutical industry and PLE has not been applied to edible seaweeds, this review attempts to provide an approximation of the PLE technique applied to seaweeds to generate knowledge that could potentially be applied in the food industry. Thus, compounds derived from edible seaweeds using PLE appear to be suitable for the bioplastic production and edible coating required in packaging; the synthesis of bio-nanocomposites that can be incorporated into food packaging to improve the mechanical properties of bioplastics; the incorporation into nutritional supplements; and the improvement of the nutritional profiles of different food products and animal feed. More experimental approaches of the PLE use for the extraction of seaweed compounds used in food products are required to determine whether this technique is appropriate for this matrix and the final products.

## Figures and Tables

**Table 1:** Bioactive compounds of microalgae, expressed as percentage dry weight, and their functional properties.

Compound		Algae	Dry Weight (%)	Functional Properties	Ref.
Polysaccharides		*Nannochloropsis oceanica*	8.33	- Prebiotic activity ^1^* - Immuno-modulatory ^1^* - Low blood sugar and lipid levels in vitro ^1^ *	[26,27,28]
		*Nannochloropsis oculata*	6.4		
		*Nannochloropsis granulata*	14.9		
		*Nannochloropsis limnetica*	10		
		*Microchloropsis salina*	17.8–36.2		
		*Microchloropsis gaditana*	21.7		
Sulfated polysaccharides		*Arthrospira platensis*	5–14.6	-Antiviral - Anti-tumor - Anti-inflammatory - Immuno-modulatory	[29,30]
		*Chlorella ellipsoidea*			
		*Phaeodactylum* sp.			
		*Schizochytrium* spp.			
		*Thraustochytrium* spp.			
Lipids		*Nannochloropsis oceanica*	18.40–46.12	-	[27,31,32,33,34,35,36]
		*Nannochloropsis oculata*	8.2		
		*Nannochloropsis granulate*	28.5		
		*Nannochloropsis limnetica*	24		
		*Microchloropsis salina*	6.2–26		
		*Microchloropsis gaditana*	16.5		
Essential fatty acids	EPA	*Nannochloropsis oceanica*	2.34	- Reduce cardiovascular disease - Improve mental health - Anti-inflammatory - Anti-diabetes - Anti-thrombotic activity	[26,27,31,32,33,34,35,36,37]
		*Nannochloropsis oculata*	2.33		
		*Nannochloropsis limnetica*	2.81		
		*Microchloropsis gaditana*	4.4–11		
		*Phaeodactylum tricornutum*	0.7–6.1 ^3^*		
		*Porosira glacialis*			
		*Monodopsis subterranean*			
		*Nannochloris* spp.			
	ARA	*Ceramium rubrum*	24–77 ^3^*	- Anti-inflammatory - Anti-cancer - Prevention of neurological disorders	[32,36]
		*Parietochloris incisa*			
		*Phormidium pseudoprsleyi*			
		*Prphyridium purpureum*			
	DHA	*Amphidiunum carterae*	17.5–30.2 ^3^*	- Decreasing preterm birth - Improving cognitive - Prevention of cardiovascular disease - Promotion of eye health - Slowing Alzheimer’s disease	[36,38]
		*Aurantochytrium* spp.			
		*Phaeodactylum tricurnutum*			
		*Schizochytrium* spp.			
		*Thraustochytrium* spp.			
Proteins		*Nannochloropsis oceanica*	14.5	- Gelling properties ^2^* - Foaming properties ^2^*	[26,27,39]
		*Nannochloropsis oculata*	22.6		
		*Nannochloropsis granulata*	45.8		
		*Nannochloropsis limnetica*	37		
		*Microchloropsis salina*	18.1–36.2		
		*Microchloropsis gaditana*	47		
Bioactive peptides		*Chlorella elipsoidea*	-	- Antihypertensive - Antibiotic - Antiviral	[40,41]
		*D. salina*			
		*Nitzschia* sp.			
		*Bellerochae* sp.			
		*Tetraselmis suecica*			
Phenolic compounds		*Chlorella vulgaris*	0.54–4.57	- Antioxidant - Anti-inflammatory - Antimicrobial - Anti-cancer - Prevention of cardiovascular and neurodegenerative diseases	[42,43]
		*Nannochloropsis* sp.			
		*Phaedactylum tricornutum*			
		*Scenedesmus obliquus*			
		*Tetraselmis* sp.			
Vitamins		*Arthrospira*	-	- Blood coagulation - Modulating inflammation - Neuroprotection, promoting eye and bone health	[44,45]
		*Chlorella*			
		*Isochrysis galbana*			
		*Pavlova*			
		*Porphyridium cruentum*			
		*Tetraselmis* sp.			

***Abbreviations:*** EPA (eicosapentaenoic acid); DHA (docosahexaenoic acid); ARA (arachidonic acid).^1^* is referred to *N. oculata* polysaccharides; ^2^* is referred to *A. platensis* proteins; ^3^* expressed as total fatty acids.

**Table 2:** Solvents used in PLE for the extraction of different compounds.

Solvent	Extracted Compound	Ref.
Water	Phenolic compounds//Di-, triterpenes//Proteins//Polysaccharides	[24,62]
Water + ionic liquids	Carrageenan//Alginates	[24,63]
Water + eutectic solvents	Carrageenan//Alginates	[24,63]
Ethanol	Polyphenols//Carotenoids//Alkaloids//Lipids	[24,64,65,66]
Aqueous ethanol	Polyphenols//Carotenoids//Alkaloids//Lipids	[24,64,65,66]
Ethyl acetate	Polyphenols//Carotenoids//Alkaloids//Lipids	[24,64,65,66]
Ethyl lactate	Fatty acids	[24,67,68]
(+)-limonene	Fatty acids	[24,67,68]

**Table 3:** Operational conditions for the PLE extraction of different compounds from seaweeds.

Seaweed	Compound	Solvent	*T* (°C)	*P* (bar)	*t* (min)	*Yield* (%)	Ref.
*Saccharina japonica (B)*	Alginate	NaOH 0.1%; H_2_Od; H_2_O	80; 110; 140	5; 25; 50	5; 12	3–27.21	[49]
	Fucoidan	NaOH 0.1%; H_2_Od; CH_2_O_2_ 0.1%	80; 110; 125; 150	5; 25; 50	5; 25	2.5–15.7	[49,62]
	TPC	H_2_O	200	50	15	39.52	
*Eisinia bicyclis (B)*	Fucoxanthin	EtOH 90%	110	103.42	5	0.39	[71]
*Laminaria ochroleuca (B)*	Fatty acids	Hexane; ethyl acetate; EtOH; EtOH 50%	120	100	10	7.42–47.16	[7]
*Sargassum muticum (B)*	Phlorotannin	EtOH- H_2_O 95:5; 75:25; 25:75.	120; 160	103.42	20	0.77–2.93	[61,72]
*Sargassum thumbergii (B)*	Phlorotannin	H_2_O	180	30	30	1.35	[73]
*Sargassum cristaelefolium (B)*	Fucoidan	H_2_O	121	1.0133	20	12.60	[74]
*Kappaphycus alvarezii (R)*	Carrageenan	H_2_O	150	50	5	71	[75]
*Ascophyllum nodosum (B)*	TPC	H_2_O; EtOH: H_2_O 60:40; EtOH: H_2_O 80:20; MeOH: H_2_O 60:40	90; 100; 120	68.95	90 s	34.5- 114	[76]
*Fucus serratus (B)*	TPC	H_2_O; EtOH: H_2_O 60:40; EtOH: H_2_O; 80:20; MeOH: H_2_O 60:40;	90; 100; 120	68.95	90 s	19.7–40.7	[76]
*Fucus vesiculosus (B)*	TPC	H2O; EtOH: H_2_O; 60:40; EtOH: H_2_O; 80:20; MeOH: H_2_O; 60:40	90; 100; 120	68.95	90 s	114.0–110	[76]
	Fatty acids	EtOH: H_2_O; 50:50	120; 160	100	10	34.85–57.19	[77]
*Ulva lactuca (G)*	Fatty acids	EtOH: H_2_O; 50:50	80; 120; 160	100	10	34.85; 41.49; 57.19	[77]
*Ulva intestinalis (G)*	Fatty acids	EtOH: H_2_O; 50:50	80; 120; 160	100	10	34.85; 41.49; 57.19	[77]
*Himanthalia elongata (B)*	Fatty acids	EtOH: H_2_O; 50:50	80; 120; 160	100	10	34.85; 41.49; 57.19	[77]

***Abbreviations:*** (G): green algae; (R): reed algae; (B): brown algae. TPC (total phenol compounds), EtOH (ethanol), MeOH (methanol), H_2_O (water), NaOH (sodium hydroxide).
